# Characterizing the Genetic Basis of Winter Wheat Rust Resistance in Southern Kazakhstan

**DOI:** 10.3390/plants14071146

**Published:** 2025-04-07

**Authors:** Shynbolat Rsaliyev, Elena Gultyaeva, Olga Baranova, Alma Kokhmetova, Rahim Urazaliev, Ekaterina Shaydayuk, Akbope Abdikadyrova, Galiya Abugali

**Affiliations:** 1 Kazakh Research Institute of Agriculture and Plant Growing, Almalybak 040909, Kazakhstan; rakhim.urazaliyev@mail.ru (R.U.); akbope81.kz@mail.ru (A.A.); g_97.02@mail.ru (G.A.); 2 All Russian Institute of Plant Protection, Shosse Podbelskogo 3, 196608 St. Petersburg, Russia; egultyaeva@gmail.com (E.G.); baranova_oa@mail.ru (O.B.); eshaydayuk@bk.ru (E.S.); 3 Institute of Plant Biology and Biotechnology, Almaty 050040, Kazakhstan; gen_kalma@mail.ru; 4 Faculty of Agrobiology, Kazakh National Agrarian Research University, Almaty 050010, Kazakhstan

**Keywords:** leaf rust, yellow rust, stem rust, molecular markers, resistance genes, wheat

## Abstract

In an effort to enhance wheat’s resilience against rust diseases, our research explores the genetic underpinnings of resistance in a diverse collection of winter bread wheat accessions. Leaf rust (*Puccinia triticina*), yellow rust (*Puccinia striiformis* f. sp. *tritici*), and stem rust (*Puccinia graminis* f. sp. *tritici*) are significant threats to global wheat production. By leveraging host genetic resistance, we can improve disease management strategies. Our study evaluated 55 wheat accessions, including germplasm from Kazakhstan, from Uzbekistan, from Russia, from Kyrgyzstan, France, and CIMMYT under field conditions in southern Kazakhstan from 2022 to 2024. The results showed a robust resistance profile: 49.1% of accessions exhibited high to moderate resistance to leaf rust, 12.7% to yellow rust, and 30.9% to stem rust. Notably, ten accessions demonstrated resistance to multiple rust species, while seven showed resistance to two rusts. Twenty accessions were selected for further seedling resistance and molecular analysis. Three accessions proved resistant to six isolates of *P. triticina*, two to four isolates of *P. striiformis*, and four to five isolates of *P. graminis*. Although no genotypes were found to be universally resistant to all rust species at the seedling stage, two accessions—Bezostaya 100 (Russia) and KIZ 90 (Kazakhstan)—displayed consistent resistance to leaf and stem rust in both seedling and field evaluations. Molecular analysis revealed the presence of key resistance genes, including *Lr1*, *Lr3*, *Lr26*, *Lr34*, *Yr9*, *Yr18*, *Sr31*, *Sr57*, and the 1AL.1RS translocation. This work provides valuable insights into the genetic landscape of wheat rust resistance and contributes to the development of new wheat cultivars that can withstand these diseases, enhancing global food security.

## 1. Introduction

Winter bread wheat (*Triticum aestivum* L.) is a crucial agricultural crop in Kazakhstan, widely cultivated in the southern and southeastern regions, including Almaty, Zhetysu, Zhambyl, and Turkestan, on both rain-fed and irrigated lands. Over the past decade, these regions have experienced significant climate changes, characterized by warmer winters and warmer, more humid springs with a slight increase in annual precipitation [1,2]. These shifts in weather patterns are marked by fluctuations both annually and monthly [3,4], which can exacerbate disease development in crops. Among the significant biotic factors affecting wheat production are rust pathogens. Leaf rust, caused by *Puccinia triticina*, yellow (stripe) rust by *P. striiformis* f. sp. *tritici*, and stem rust by *P. graminis* f. sp. *tritici*, are major threats to wheat yields. Understanding and addressing these challenges is crucial for maintaining the health and productivity of wheat crops in Kazakhstan.

In Kazakhstan, leaf rust is a prevalent issue affecting wheat crops [5,6,7,8], with epidemics occurring approximately every 4 years. This frequency is linked to an expansion in wheat cultivation [9]. The leaf rust population in Kazakhstan is genetically diverse, as evidenced by the wide range of virulence among studied races. Recent studies have identified 25 different races of wheat leaf rust in the country. An analysis using 16 *Lr* lines revealed diverse virulence types, ranging from less virulent strains like “CJF/B” and “JCL/G” to highly virulent ones such as “TKT/Q”. Most pathotypes were avirulent to *Lr9*, *Lr19*, *Lr24*, and *Lr25* but virulent to *Lr1*, *Lr2a*, *Lr3ka*, *Lr11*, and *Lr30* [7]. Kazakhstani wheat cultivars and lines primarily contain leaf rust-resistance genes such as *Lr1*, *Lr9*, *Lr10*, *Lr19*, *Lr26*, *Lr34*, *Lr37*, *Lr46*, and *Lr68*, often in combination [7,10]. The most frequently detected genes were *Lr37*, *Lr34*, and *Lr46*, while *Lr19*, *Lr68*, *Lr26*, and *Lr28* were less common. Some cultivars, like Keremet and Hisorok, carried four *Lr* genes, while others, including Aliya, Rasad, Reke, Mataj, Egana, and Almaly/Obri, carried three. Molecular screening identified 29 carriers of one *Lr* gene, 10 with two genes, 6 with three genes, and 2 with four genes. The combination of *Lr37*, *Lr34*, and *Lr68* proved particularly effective, as carriers showed a low susceptibility index to diseases [8].

Yellow rust is a prevalent disease affecting winter wheat across Central Asia, including Kazakhstan. In favorable years, it often leads to widespread infection in crops [9,11,12,13,14,15]. This disease has become a significant constraint on winter wheat production in the region [9,11,12,16]. Historical data from Kazakhstan, Kyrgyzstan, and Uzbekistan indicate that the yellow rust population in Central Asia shares genetic similarities with populations in Western Asia [17]. In Central Asia, yellow rust typically exhibits high virulence to certain resistance genes, such as *YrA*, *Yr2*, *Yr6*, *Yr7*, *Yr9*, and *Yr25*. However, it is less virulent or not virulent to others like *Yr5*, *Yr10*, *Yr15*, *Yr24*, and *YrSp* [18].

In addition, due to the emergence and spread of the Ug99 virulent race, which creates devastating epidemics in the world [19,20,21,22], stem rust has become relevant for the regions of spring and winter wheat cultivation in Kazakhstan [9,23,24,25]. Currently, new virulent races of stem rust continue to endanger grain crops in the Central Asian region, particularly in Kazakhstan [26,27,28].

Farmers of Kazakhstan cultivate mainly local winter wheat cultivars Almaly, Bogarnaya 56, Zhetysu, Mereke 70, Sapaly, Steklovidnaya 24, Farabi, and others. These cultivars are notable for their high drought tolerance and adaptability to diverse environmental conditions. Some of these cultivars also exhibit field resistance to rust species. For instance, the Almaly cultivar contains the resistance genes *Lr34/Yr18* [26,29], while Bogarnaya 56 carries *Lr3a* and *Lr13* [30]. The Zhetysu cultivar also has *Lr13* [30], Mereke 70 contains *Yr10* and *Yr18/Lr34* [29], and Sapaly has *Lr3* along with powdery mildew resistance genes *Pm3c* and *pm8* [31]. Steklovidnaya 24 is equipped with *Lr3*, *Lr13*, *Pm3c*, and *pm8* [30,31]. Although many identified resistance genes, except for the *Lr34/Yr18* complex and *Yr10*, have limited effectiveness both in Kazakhstan and globally, most domestic winter wheat cultivars are tolerant to rust damage. This tolerance means that despite moderate rust development, these cultivars generally do not experience significant reductions in grain productivity. It is possible that secondary resistance genes play a role in this resilience.

Kazakhstani and Russian researchers have employed molecular screening to identify promising wheat lines that possess a complex of rust-resistance genes. For instance, the *Lr35/Sr39* gene complex was identified in wheat lines 304/14 and 125/14, while line 351/12 carried *Lr37/Yr17/Sr38*. Lines 89/14 and 386/13 were found to have both *Lr35/Sr39* and *Lr37/Yr17/Sr38*. Additionally, lines 362/13, 116-10-4, and 211-10-10 contained *Lr35/Sr39*, and lines 239-10-17 and 56-10-13 had *Lr37/Yr17/Sr38*. Some lines, such as 319/14, 129/12, 366-13-5, and 385/12, were identified with multiple gene complexes, including *Lr35/Sr39* and *Lr37/Yr17/Sr38* [29]. In Kazakhstan, attractive gene combinations for rust resistance include *Lr19/Sr25*, *Lr26/Sr31/Yr9/Pm8*, *Lr34/Yr18 APR*, and *Lr37/Yr17/Sr38*. These combinations are proposed for use in developing wheat cultivars resistant to multiple rust species [29,32,33]. A study of 70 domestic winter wheat genotypes for yellow rust resistance found that 15 cultivars and 27 breeding lines were resistant. Molecular marker analysis revealed the presence of several genes and gene complexes, including *Yr5*, *Yr10*, *Yr15*, *Yr17/Lr37/Sr38*, and *Yr18/Lr34*. The *Yr10* gene was the most common, identified in 22 genotypes, followed by *Yr5* in 14 lines (20%), and *Yr18* in 11 lines (15.7%). *Yr15* was found in seven lines and the *Yr17/Lr37/Sr38* complex in two genotypes [14].

Combining multiple resistance genes in a single wheat genotype can significantly enhance its defense against various diseases. For instance, integrating genes like *Sr2/Lr27/Yr30* and *Lr34/Yr18/Sr57* can substantially improve resistance to all three species of rust [34]. Strategically utilizing these pleiotropic genes, which confer resistance to multiple pathogens, in combination with other secondary genes is recommended for breeding rust-resistant wheat cultivars. From a practical standpoint, it is crucial to strike a balance between disease resistance and plant growth. While accumulating multiple resistance genes can enhance protection, it can also divert substantial energy from the host plant, potentially reducing yields [35]. Therefore, breeders must carefully manage the integration of these genes to ensure that the benefits of increased resistance do not compromise overall plant productivity.

Utilizing carriers of various minor *Yr*, *Lr*, and *Sr* genes can enhance the resistance of winter wheat to yellow, leaf, and stem rust in breeding programs. Modern wheat breeding techniques offer an effective approach to creating cultivars with strategic combinations of genes, which can contribute to developing durable resistance in the region. The objective of these studies was to assess the resistance of winter wheat cultivars and lines to leaf, yellow, and stem rust at both the adult plant and seedling stages. Additionally, the goal was to identify the genetic characteristics of resistant host plants in southern Kazakhstan, providing valuable insights for future breeding initiatives.

## 2. Results

### 2.1. Adult Plant Resistance Test

A collection of 55 promising winter bread wheat genotypes was assessed for rust resistance under field conditions in southern Kazakhstan from 2022 to 2024 (Table 1).

The evaluation revealed varying levels of resistance to leaf rust among the accessions. Notably, two Kazakhstani accessions, KIZ 90 and 20403-2, demonstrated high immunity to leaf rust, with no disease severity observed. Two Russian cultivars, Akhmat and Bezostaya 100, showed minimal infection, characterized by necrotic flecks with single pustules. Thirteen accessions exhibited moderate resistance, with disease severity ranging from 5% to 10%, while eighteen accessions displayed moderate susceptibility, with severity between 20% and 30%. The remaining genotypes had disease severity between 40% and 60%, which was lower than that of the susceptible standard, which had a severity of 80%.

In the assessment of yellow rust resistance, seven accessions (12.7%) displayed varying levels of resistance. Notably, two Kazakh lines, 21730-1 and 22372K, showed no symptoms of yellow rust (DS: 0%). Moderate resistance (DS: 5–20%) was observed in 14 accessions, while 11 accessions exhibited moderate susceptibility (DS: 20–30%). For other winter wheat genotypes, yellow rust severity ranged from 40% to 80%, with the susceptible standard being affected at 80%.

Different levels of stem rust resistance were identified in 17 accessions (30.9%). Kazakh cultivar KIZ 90 and lines 21730-1, 22372K, demonstrated a high level of field resistance to stem rust. Eleven accessions showed moderate resistance (DS: 5–20%) and twenty-three accessions displayed moderate susceptibility (DS: 20–30%). Two cultivars, Arap and Bakytzhan, and two lines, 20521-1 and 21203-11-3, were affected similarly to the susceptible standard, with disease severity ranging from 40% to 60%.

Ten accessions were found to be resistant or moderately resistant to all three types of rust. Cultivars KIZ 90, Alekseyich, Akhmat, Bezostaya 100, and lines 21730-1, 22372K exhibited very high resistance to leaf, yellow, and stem rust. Four genotypes—Adilet, 18410-1, 20197-17, and 22180-1—showed moderate resistance to the three rust types (disease severity: 10–20%). However, no accessions were completely immune (DS: 0%) to all three rust species.

Seven accessions were moderately resistant to two rust species. Cultivars Mereke 70 and Ilgor showed resistance to leaf and yellow rust, while lines 20403-2, 21144-4-1, 22315-1, 22353K, and D68CIMMYT were resistant to leaf and stem rust.

### 2.2. Seedling Rust-Resistance Test

Seedling infection type data for 20 promising winter wheat accessions inoculated with *P. triticina*, *P. graminis*, and *P. striiformis* pathotypes are presented in Table 2.

Leaf Rust. Only three accessions—Bezostaya 100, KIZ 90, and 18410-1—were found to be resistant to six *P. triticina* isolates and all of these accessions also showed resistance to leaf rust in field conditions. Notably, more accessions were resistant to Kazakh isolates of *P. triticina* compared to Russian ones. The leaf rust isolates used in the study varied in their virulence to several resistance genes, including *Lr1*, *Lr2a*, *Lr2b*, *Lr2c*, *Lr9*, *Lr15*, *Lr19*, and *Lr26*. A multi-pathogen test identified the *Lr26* gene in three wheat accessions: Akhmat and lines 21730-1 and 22372K. These genotypes were resistant to PtK1-PtK4 isolates, which are avirulent to *Lr26*, but susceptible to the virulent PtK5 and PtK6 isolates. The tests revealed the absence of the *Lr9* and *Lr19* genes in the studied wheat collection. No accessions were found that were susceptible only to the PtK3 or PtK4 isolate and resistant to the other isolates tested. Additionally, the absence of the *Lr2a*, *Lr2b*, *Lr2c*, and *Lr15* genes was confirmed. The *P. triticina* isolate PtK6 was distinct from others due to its avirulence to the *Lr2a*, *Lr2b*, *Lr2c*, and *Lr15* genes. However, no accessions were identified that were resistant to the PtK6 isolate and susceptible to the other isolates tested.

High (R) and moderate resistance (MR) to stem rust was observed in three Kazakh lines—KIZ 90, 21730-1, and 22372K—and one Russian cultivar, Bezostaya 100. All these genotypes demonstrated resistance to stem rust under field conditions. The *P. graminis* isolates varied in their virulence to several resistance genes, including *Sr7b*, *Sr8a*, *Sr9e*, *Sr9b*, *Sr11*, *Sr17*, *Sr24*, *Sr30*, *Sr36*, and *SrTmp*. Notably, Kazakh isolates (PgK1 and PgK2) had fewer virulence alleles compared to Russian isolates. Cultivar Adilet and lines 18410-1, 20521-1, 21203-11-3, and 22353K were resistant to two Kazakh *P. graminis* isolates (PgK1 and PgK2). Additionally, cultivars Akhmat, Euclide, and SWW1-904 were resistant to the PgK2 isolate, while line 22180-1 was also resistant to PgK2. These isolates differed in their avirulence to the *Sr7b*, *Sr9e*, *Sr9b*, *Sr36*, and *SrTmp* genes. The multi-pathogen tests revealed the absence of the *Sr24* gene in the studied wheat collection. No accessions were found that were susceptible only to the PgK4 isolate and resistant to the other isolates tested.

The number of accessions resistant to yellow rust was notably lower compared to those resistant to stem rust and leaf rust. Only two accessions, Akhmat and line 18410-1, showed resistant reactions to all *P. striiformis* isolates. However, these accessions displayed a moderate level of resistance to yellow rust under field conditions. Resistance to two Kazakh isolates of *P. striiformis* (Pst_1, Pst_2) was observed in 11 wheat accessions, while four accessions were resistant to only one of these isolates. Among them, six accessions—22372K, 22353K, 21203-11-3, 20197-17, KIZ 90, and Bezostaya 100—demonstrated resistance in the field. The number of genotypes resistant to Kazakh isolates (Pst_1 and Pst_2) was higher than those resistant to Russian isolates (Pst_3 and Pst_4), although the isolates did not differ significantly in virulence. All isolates were avirulent to *Yr5*, *Yr10*, and *YrSP*, but virulent to *Yr8*. The main difference among them was their virulence or avirulence to genes *Yr1*, *Yr7*, *Yr9*, and *Yr24* (=*Yr26*). Following multi-pathogen testing, the *Yr9* gene was identified in line 21730-1, which was resistant to the Pst_1 isolate avirulent to *Yr9*, but susceptible to other isolates with virulence to this gene. No other *Yr* genes were detected in the wheat accessions tested during the multi-pathogen test.

Overall, no lines were identified that were resistant to all rust species in the seedling resistance study. However, two accessions, Bezostaya 100 and KIZ 90, showed seedling resistance to both leaf and stem rust and were also resistant in the field.

### 2.3. Identification of Rust-Resistance Genes Using Molecular Markers

Molecular markers were utilized to identify a range of leaf rust-resistance genes, including *Lr1*, *Lr3*, *Lr9*, *Lr10*, *Lr19*, *Lr20*, *Lr24*, *Lr25*, *Lr26*, *Lr28*, *Lr29*, *Lr34*, *Lr37*, *Lr41(39)*, *Lr47*, *Lr51*, *LrAsp*, and *Lr6Agi2*. Additionally, yellow rust-resistance genes *Yr9*, *Yr17*, and *Yr18*, as well as stem rust-resistance genes *Sr2*, *Sr15*, *Sr24*, *Sr25*, *Sr26*, *Sr28*, *Sr31*, *Sr36*, *Sr38*, *Sr39*, and the 1AL.1RS translocation, were identified.

In the studied collection, specific leaf rust-resistance genes detected included *Lr1*, *Lr3*, *Lr26*, and *Lr34*. Yellow rust-resistance genes identified were *Yr9* and *Yr18*, while stem rust-resistance genes included *Sr31* and *Sr57*, along with the 1AL.1RS translocation. The most frequently identified rust genes, either alone or in combination, were *Lr34*, *Yr18*, and *Sr57*, found in eight accessions. The *Lr3* gene was postulated in five accessions and the *Lr1* gene in four accessions. The 1BL.1RS rye translocation, carrying the *Lr26*, *Yr9*, and *Sr31* genes, was detected in four accessions, while the 1AL.1RS rye translocation was found in one cultivar (Table 2).

## 3. Discussion

Rust species are a significant challenge in wheat production in Kazakhstan, where epidemics typically occur every 4 years. In years with sufficient rainfall in April and May, diseases spread rapidly among winter wheat crops in the south and southeast of the country. Currently, there is limited information on the resistance of wheat cultivars from Central Asia at both the adult plant and seedling stages. To address this gap, our study aimed to evaluate the resistance of adult plants in the field and wheat seedlings in controlled environments. Additionally, we sought to identify the primary and secondary *Lr*, *Yr*, and *Sr* genes responsible for resistance to leaf, yellow, and stem rust, respectively. Our research followed the recommendations of prominent scientists to breeders, phytopathologists, and geneticists working in Central Asia. We assessed a collection of wheat cultivars in field hotspots and in greenhouses to determine seedling resistance to specific rust races. This approach helps identify cultivars that demonstrate resistance to diseases, which can then be transferred to susceptible but locally adapted cultivars through simple crosses. Any breeding program in the region can utilize this straightforward breeding methodology to achieve long-term rust control [36].

In the southeast of Kazakhstan, the years 2022 and 2024 were conducive to the development of rust species in the field, with a rainy and cool spring contributing to the strong spread of leaf, yellow, and stem rust on susceptible cultivars. Yellow rust was particularly prevalent among the studied genotypes, highlighting its relevance in southern Kazakhstan and throughout Central Asia compared to leaf and stem rust [9,11,12,16]. When evaluating the resistance of winter wheat against this infectious backdrop, several cultivars and lines stood out for their resistance to all three rust species. Cultivars KIZ 90 (Kazakhstan), Alekseyich, Akhmat, Bezostaya 100 (Russia), and breeding lines 21730-1 and 22372K were among the most resistant. Four genotypes—Adilet, 18410-1, 20197-17, and 22180-1—displayed moderate resistance to the three rust species. However, no samples were found to be completely immune to all three rust species. Seven samples were moderately resistant to two rust species: cultivars Mereke 70 and Ilgor to leaf and yellow rust, and lines 20403-2, 21144-4-1, 22315-1, 22353K, and D68CIMMYT to leaf and stem rust. The resistance of these cultivars and lines was generally categorized as moderate resistance. Notably, genotypes Bezostaya 100, KIZ 90, 21730-1, and 22372K showed an immune response to the stem rust population in field conditions (Table 1) and to individual races in greenhouse conditions (Table 2).

In our research, we focused not only on primary resistance genes with significant effects but also on secondary genes, particularly those that confer resistance to multiple rust species. This approach is crucial for ensuring long-term resistance, as combining several minor genes can provide a level of “near immunity” to rust diseases in wheat [37]. Additionally, integrating multiple resistance genes involved in various defense mechanisms enhances resistance to different rust species. For instance, combining *Lr34/Yr18/Sr57* significantly improves resistance to all three types of rust [34]. Our molecular genetic study of 20 winter wheat genotypes used molecular markers to identify 18 *Lr* genes, 3 *Yr* genes, and 10 *Sr* genes, as well as to determine the 1AL.1RS translocation. The results showed that in eight genotypes, resistance to the three rust species is attributed to a combination of the *Lr34*, *Yr18*, and *Sr57* genes (Table 2). This gene complex provides partial and race-independent resistance to a variety of pathogens, including all three types of wheat rust. Currently, the *Lr34/Yr18/Sr57* gene complex is widely utilized in breeding programs. Leaf Tip Necrosis in wheat serves as a visual marker for this gene complex, facilitating selection [38]. The *Yr18* gene is noted for providing long-term stability, contributing to grain yields ranging from 36% to 58%, depending on the year and sowing date, even with a significant prevalence of yellow rust [39]. In Egyptian wheat cultivars like Sakha 94 and Shandaweel 1, which exhibit slow yellow rust development in adult plants, the presence of the *Yr18* gene associated with Adult Plant Resistance has been confirmed using phenotypic and genotypic markers [40]. Furthermore, the *Lr34* gene, combined with other secondary additive genes, is effective in providing long-term resistance to leaf rust in wheat cultivars during adult plant growth [41,42]. Overall, the *Lr34*, *Yr18*, and *Sr57* genes have been instrumental in enhancing rust resistance globally, but ongoing efforts are needed to maintain their effectiveness against evolving rust populations.

Another significant gene complex, comprising *Lr26*, *Yr9*, and *Sr31*, has been extensively utilized in wheat breeding programs [43]. This complex was introduced into the wheat genome through wheat-rye translocation chromosomes. Using molecular markers, we identified these genes in the genotypes of the Bezostaya 100 and KIZ 90 cultivars, as well as in breeding lines 21730-1 and 22372K. Although the *Sr31* resistance gene has lost effectiveness in regions where Ug99 racial variants are prevalent, cultivars carrying this gene still exhibit resistance to stem rust in many countries [9,19,20,44]. We also detected a 1AL.1RS rye translocation in the Akhmat cultivar. A similar translocation was found in a CIMMYT line (identification number 8248316). In both the Akhmat cultivar and this CIMMYT line, stem rust severity was limited to 5% or less [45]. This highlights the ongoing utility of these genetic resources in maintaining resistance to stem rust.

Winter wheat cultivars and breeding lines selected in southern Kazakhstan, which contain the *Lr34*, *Yr18*, *Sr57*, as well as *Lr26*, *Yr9*, and *Sr31* gene complexes, are highly valuable for providing partial and long-term resistance to rust species. When combined with other primary and secondary genes, these complexes can effectively inhibit the emergence of virulent races of leaf, yellow, and stem rust. Our findings align with those reported by other researchers, highlighting the importance of these gene combinations in enhancing rust resistance [34,46,47,48,49,50,51,52,53].

## 4. Materials and Methods

### 4.1. Plant Material

The field rust-resistance study involved 55 advanced winter wheat genotypes (*Triticum aestivum* L.), comprising 35 from Kazakhstan, 7 from Uzbekistan, 5 from Russia, 3 from Kyrgyzstan, 1 from France, and 4 from CIMMYT. Twenty promising accessions, including fifteen from Kazakhstan, two each from Russia and CIMMYT, and one from France, were selected for seedling resistance and molecular studies (Table S1).

### 4.2. Assessment of Field Response to Leaf, Yellow, and Stem Rust

In the experimental field nursery of the Kazakh Research Institute of Agriculture and Plant Growing (N 43°13′09″ E 76°41′17″), experiments were conducted to study the resistance of winter wheat to rust species. For this purpose, the seeds of winter wheat cultivars and lines were sown using the standard method (1-m row with a distance between rows of 20 cm). The susceptible standards (cultivars Bogarnaya 56 for leaf rust, Morocco for yellow rust, and Bakytzhan for stem rust), which were spreader rows of infection, were used as a susceptible indicator. Seeds of their cultivars were sown every 20 rows to ensure uniform spread of leaf, yellow, and stem rust infection. Inoculation of the studied genotypes was carried out with a *P. triticina*, *P. striiformis*, and *P. graminis* population with talc in a ratio of 1:100, with a load of 20 mg of spores per sq. m. The inoculum of the each of population was a mixture of uredospores from a local population of the fungus, previously collected from wheat crops in the experimental area. The first assessment of the development of the disease was carried out at the beginning of its manifestation, subsequent ones—with an interval of 10–12 days until the milky-waxy ripeness of the grain. The main phytopathological parameters for assessing genotypes for resistance to the rust pathogens were: infection type (IT) and disease severity (DS, %). The IT was determined using the recommended CIMMYT score [54]: I (immune), R (resistant), MR (moderately resistant), MS (moderately susceptible), S (susceptible). The DS of plants (%) was determined using the modified Cobb scale [55]. The coefficient of infection (CI) for rust development was calculated by multiplying the constant values of IT by the DS. Constant values of infection types were used: I = 0.0; R = 0.2; MR = 0.4; M = 0.6; MS = 0.8 and S = 1.0 [56]. The CI value is used for grouping accessions by resistance to rust species; a CI in the range of 0–2.0 means highly resistant genotypes, 8.0–24.0 are moderate resistant, 40.0 or more belongs to the susceptible group.

Weather conditions in 2022 and 2024 were more favorable for rust development than in 2023. The annual precipitation for 3 years ranged from 469.0 to 758.9 mm (Table 3).

Weather conditions in Almaty region in 2022 were moderately favorable for the development of three rust species. On susceptible standards, the maximum severity of leaf, yellow, and stem rust were 60%, 80%, and 40%, respectively (Table 3).

In 2023, insufficient rainfall in May (43.4 mm) and especially in June (4.3 mm) resulted in disease depression on wheat. No symptoms of yellow rust and stem rust were detected. Moderate development of leaf rust was observed for eight accessions: 20933-1, 20982-2 (DS: 5%), Almaly, Dulaty, Egemen, Steklovidnaya 24, Vavilov, D580CIMMYT (DS: 10%).

Weather conditions in 2024 were highly favorable for the development of three species of rust. In April, the average monthly air temperature was 12.8 °C and the total precipitation was 111.3 mm. In May, the average air temperature was 17.6 °C and the sum of precipitation was 121.2 mm, which is 1.2 times more than the multi-year indicator. Cool night temperatures with an average of 12.4 °C favored the successful development of yellow rust. Leaf and yellow rust severity on susceptible standards reached 80% and stem rust 60%.

### 4.3. Seedling Rust-Resistance Tests

Two research centers carried out seedling resistance studies (Kazakh Research Institute of Agriculture and Plant Growing, Almalybak, Kazakhstan) and All-Russian Institute of Plant Protection, St. Petersburg, Russia).

Leaf rust infection at the seedling stage was evaluated for six *P. triticina* isolates with different virulence/avirulence combinations, yellow rust infection for four *P. striiformis* isolates, and stem rust infection for five *P. graminis* isolates. For leaf rust, Thatcher’s isogenic lines (Tc*Lr*) with genes *Lr*: 1, 2a, 2b, 2c, 3a, 3bg, 3ka, 9, 11, 14a, 15, 16, 17, 20, 24, 26, 29, and 30 were used in both laboratories. In addition, the Tc*Lr25* line was added in Kazakhstan to characterize the virulence of *P. triticina* isolates and Tc*Lr10*, Tc*Lr14b*, Tc*Lr18*, and Tc*Lr28* lines and breeding accessions with *Lr47* and *Lr51* were added in Russia [57]. Based on the North American system of nomenclature [58]. Kazakh isolates Pt_1 and Pt_2 belonged to the TGT and KHT races and Russian isolates PtK1-K4 to TLT, TGT, THT, and MHT races correspondingly. Virulence/avirulence was determined with the following differential sets: group I: *Lr1*, *Lr2a*, *Lr2c* and *Lr3a*; group II: *Lr9*, *Lr16*, *Lr24* and *Lr26*; group III: *Lr3ka*, *Lr11*, *Lr17* and *Lr30*.

Avocet lines with genes *Yr*: 1, 5a, 6, 7, 8, 9 10, 15, 17, 24, 27, and 5b (=*YrSp*) and 15 cultivars from International (Chinese 166, Lee, Heines Kolben, Vilmorin 23, Moro, Strubes Dickkopf, Suwon 92/Omar) and European (Hybrid 46, Reicherberg 42, Heines Peko, Nord Desprez, Compair, Carstens V, Spalding Prolific, Heines VII) differential sets were used for determination of virulence of *P. striiformis* isolates used in multi-pathogen test [59]. The infection type (IT) of wheat seedlings to yellow rust was determined according to the Gassner and Straib scale [60].

Designation of races for *P. graminis* was made using the following international differential sets; group I: *Sr5*, *Sr21*, *Sr9e* and *Sr7b*; group II: *Sr9a11*, *Sr6*, *Sr8a* and *Sr9g*; group III: *Sr36*, *Sr9b*, *Sr30* and *Sr17*; group IV: *Sr9a*, *Sr9d*, *Sr10* and *SrTmp*; and group IV: *Sr24*, *Sr31*, *Sr38*, and *SrMcN* [61]. Accordingly, Kazakh isolates PgK1 and PgK2 were designated as THMTF and QHHSF races and Russian isolates PgK3-K5 as TTTTF, TTTTP, and TTSTF races (Table 4).

For evaluation of seedling resistance to three rust species seeds of 20 studied wheat seeds accessions (from 5 to 10 seeds) were sown in plastic pots with a volume of 200 mL. Spores of a single-pustule isolates each rust species were mixed with a light mineral oil (NOVEC 7100) and sprayed onto the 10-day old wheat plants (seedlings with the first leaf fully unfolded) for leaf and stem rust-resistance studies and 12–14-day old plants (second leaf appearance) for yellow rust-resistance study. Wheat seedlings inoculated by *P. triticina* and *P. graminis* isolates, were incubated in a dark humid chamber for 16–24 h at 20–22 °C, and inoculated by *P. striiformis* isolates at 10 °C. Then wheat plants were incubated in greenhouse boxes with photoperiod 16 h day (10–15 thousand lux)/8 h night at 20–22 °C for leaf and stem rust. The plants inoculated by yellow rust isolates transferred to a growth chamber (Environmental Test Chamber MLR-352H, Sanyo Electric Co., Ltd., Osaka, Japan) with 16:8 h L:D photoperiod at 16 and 10 °C, respectively. Infection types were assessed 12 days after the incubation for leaf and stem rust (Mains and Jackson and Stakman and Levin scales correspondingly) [62,63] and after 16–18 days for yellow rust (Gassner and Straib scale) on a five-point scale [60]. Isolates with infection types 0–2 and 3–4 were assumed to be avirulent and virulent, respectively.

### 4.4. Identification of Lr, Sr, and Yr Genes Using Molecular Markers

Identification of rust-resistance genes was carried out at the All-Russian Institute of Plant Protection (St. Petersburg). Molecular markers were used for the identification of 18 *Lr* genes (*Lr1*, *Lr3*, *Lr9*, *Lr10*, *Lr19*, *Lr20*, *Lr24*, *Lr25*, *Lr26*, *Lr28*, *Lr29*, *Lr34*, *Lr37*, *Lr41(39)*, *Lr47*, *Lr51*, *LrAsp*, and *Lr6Agi2*), 3 *Yr* genes (*Yr9*, *Yr17* and *Yr18*), 10 *Sr* genes (*Sr2*, *Sr15*, *Sr24*, *Sr25*, *Sr26*, *Sr28*, *Sr31*, *Sr36*, *Sr38*, *Sr39*), and 1AL.1RS translocation carries the stem rust-resistance gene *SrR*, but no known leaf and yellow resistance genes [64]. The list of used molecular markers of *Lr*, *Yr*, and *Sr* genes is presented in Table 5.

In the leaf and yellow rust study, DNA was extracted according to Dorokhov and Cloquet [85]. PCRs were performed using a thermocycler (C1000, BioRad, Hercules, CA, USA). PCR mixture (20 mL) contained 100–150 ng of genomic DNA, 2 units of Taq DNA polymerase, 1× PCR buffer (10 mM Tris HCL), 2.5 mM of MgCl_2_, 100 mM of each dNTP, and 10 mM of each primer. The recommended PCR protocol (Table 5) was used in amplifications. In stem rust study, the PCR mixture BioMaster HS-Taq PCR-Color (BIOLABMIX LLC, Novosibirsk, Russia) and the following amplification conditions were applied: 95°—5 min, 35 cycles (95°—20 s, annealing temperature—30 s, 72°—1 min), and 72°—5 min was used. The annealing temperature was individual for each pair of primers. For the *Sr2* gene marker, csSr2, the following PCR mixture was used: 20 µL of the reaction mixture; bidistilled H_2_O—17.6 µL; a mixture of dNTPs (25 mM)—0.4 µL; primer R (10–15 pmol)—0.5 µL; primer F (10–15 pmol)—0.5 µL; 10× PCR buffer—2.5 µL; MgCl_2_ (50 mM)—1 µL; Taq polymerase (5U)—0.5 µL; and genomic DNA—2 µL. The amplification conditions were as follows: 94°—4 min 30 s, 45 cycles (94°—1 min, 60°—1 min, 72°—2 min), 72°—10 min. After PCR, the amplification products were treated with restriction endonuclease BspHI. PCR products were separated on 1.5 to 3.0% agarose gels (depending on gene product size) and visualized under UV light using the digital gel imaging system (GelDocGo, BioRad, Hercules, CA, USA).

## 5. Conclusions

In our research, a collection of 55 winter wheat cultivars and breeding lines in southern Kazakhstan exhibited diverse reactions to natural populations of leaf, yellow, and stem rust. During the favorable disease development conditions in 2022 and 2024, 20 wheat cultivars and lines with Adult Plant Resistance were selected. Using specific virulent races from Kazakhstan and Russia, the resistance of 12 genotypes at the seedling stage was confirmed. Molecular methods identified eight sources carrying the *Lr34*, *Yr18*, and *Sr57* genes, four sources with *Lr26*, *Yr9*, and *Sr31*, as well as sources with *Lr1*, *Lr3*, and the 1AL.1RS translocation. The utilization of winter wheat cultivars containing secondary resistance genes is highly valuable for providing partial and long-term resistance to leaf, yellow, and stem rust. This approach helps mitigate the emergence of new virulent races in the region, contributing to sustainable wheat production.

## Figures and Tables

**Table 1:** Rust infection type, disease severity, and coefficient of infection of winter wheat accessions in the field in Southern Kazakhstan in 2022–2024.

No.	Entry	Rust Infection Type (IT), Disease Severity (DS), and Coefficient of Infection (CI)								
		*P. triticina*			*P. striiformis* f. sp. *tritici*			*P. graminis* f. sp. *tritici*		
		IT *	DS, % **	CI ***	IT *	DS, % **	CI ***	IT *	DS, % **	CI ***
1.	Adilet	MR	20	8.0	MR	20	8.0	R	5–10	2.0
2.	Almaly	MR	10–20	8.0	MR	20	8.0	MR	10–20	8.0
3.	Amanat	MS	20–30	24.0	S	40–60	60.0	MS	20–30	24.0
4.	Arap	MS	20–30	24.0	MR-MS	20–30	24.0	S	40	40.0
5.	Bakytzhan	MS	20–30	24.0	MR-MS	20–30	24.0	S	40–60	60.0
6.	Dimash	MS	20–30	24.0	MR	10–20	8.0	MS	20–30	24.0
7.	Dulaty	MR	10–20	8.0	R-MR	5–10	4.0	MR	10–20	8.0
8.	Egemen 20	MS-S	30–40	40.0	MS-S	30–40	40.0	MS	20–30	24.0
9.	Farabi	MS-S	30–40	40.0	MS	20–30	24.0	MS	20–30	24.0
10.	Kazakhstan 10	MS-S	30–40	40.0	MR-MS	20–30	24.0	MR-MS	20–30	24.0
11.	KIZ 90	I	0	0.0	I-R	0–5	1.0	I	0	0.0
12.	Mereke 70	R-MR	5–20	8.0	R	5–10	2.0	MR-MS	20–30	24.0
13.	Momyshuly	MS	20–30	24.0	MR-MS	20–30	24.0	MR-MS	20–30	24.0
14.	Nesipkhan	MS	20–30	24.0	MR	10–20	8.0	MR-MS	20–30	24.0
15.	Pamyat 47	MS	20–30	24.0	S	60–80	80.0	MR-MS	20–30	24.0
16.	Sapaly	MR-MS	20–30	24.0	MR-MS	10–30	24.0	R-MR	5–10	4.0
17.	Steklovidnaya 24	MS-S	30–40	40.0	MS-S	30–40	40.0	MS	20–30	24.0
18.	Talimi 80	MR-MS	20–30	24.0	MR	10–20	8.0	MR	10–20	8.0
19.	Vavilov	MS-S	30–40	40.0	MR-MS	20–30	24.0	MR-MS	20–30	24.0
20.	Zhetysu	MS-S	30–40	40.0	MS-S	30–40	40.0	MS	20–30	24.0
21.	18410-1	R-MR	5–20	8.0	R-MR	5–20	8.0	MR	10–20	8.0
22.	18411-1	MS	20–30	24.0	MS-S	30–40	40.0	R	5–10	2.0
23.	20197-17	R-MR	5–20	8.0	R-MR	5–20	8.0	R-MR	5–20	8.0
24.	20403-2	I	0	0.0	MR-MS	20–30	24.0	R	1–5	1.0
25.	20521-1	MS	30	24.0	S	40	40.0	S	40	40.0
26.	20933-1	MS	20–30	24.0	MR-MS	20–30	24.0	R-MR	5–20	8.0
27.	20982-2	MS	20–30	24.0	R-MR	5–10	4.0	MR	10–20	8.0
28.	21144-4-1	R	5–10	2.0	MR-MS	20–30	24.0	R-MR	5–10	4.0
29.	21203-11-3	MS	20–30	24.0	S	40	40.0	S	40	40.0
30.	21266-3	MR	10–20	8.0	MR	10–20	8.0	R-MR	5–10	4.0
31.	21730-1	R	5–10	2.0	I	0	0.0	I	0	0.0
32.	22180-1	MR	10–20	8.0	MR	10–20	8.0	MR	10–20	8.0
33.	22315-1	R	5–10	2.0	MR-MS	20–30	24.0	I-R	0–5	1.0
34.	22353K	R-MR	1–20	8.0	MR	10–20	24.0	R	1–10	2.0
35.	22372K	R	1–5	1.0	I	0	0.0	I	0	0.0
36.	Alekseyich	R	1–10	2.0	I-R	0–5	0.5	R	1–5	1.0
37.	Akhmat	I-R	0–1	0.2	R	1–5	1.0	R	1–5	1.0
38.	Bezostaya 100	I-R	0–1	0.2	R	1–10	2.0	I	0	0.0
39.	Grom	R	1–10	2.0	MR	10–20	8.0	MS	20–30	24.0
40.	Gurt	MS	20–30	24.0	S	60–80	80.0	MR	10–20	8.0
41.	Bardosh	MS	20–30	24.0	MR	5–20	8.0	MR-MS	10–30	24.0
42.	Ezoz	R	1–10	2.0	S	60–80	80.0	MR-MS	20–30	24.0
43.	Ilgor	R	1–10	2.0	R	5–10	2.0	MR-MS	10–30	24.0
44.	Kayraktosh	R	1–10	2.0	MS-S	30–60	60.0	MR-MS	10–30	24.0
45.	Ok marvarid	MS-S	30–40	40.0	MS-S	30–40	40.0	MR-MS	20–30	24.0
46.	Pahlavon	R-MR	5–20	8.0	MS-S	30–40	40.0	MR-MS	20–30	24.0
47.	Tespishar	MS	20–30	24.0	S	60–80	80.0	MR	10–20	8.0
48.	Ajara	MR	20	8.0	S	60–80	80.0	MR-MS	10–30	24.0
49.	Asyl	MR	20	8.0	MR-MS	10–30	24.0	MR-MS	20–30	24.0
50.	Intensivnaya	MS-S	30–60	60.0	MS-S	30–40	40.0	MR-MS	10–30	24.0
51.	D68CIMMYT	R	5–10	2.0	S	40	40.0	R-MR	5–10	4.0
52.	D580CIMMYT	MS-S	30–60	60.0	S	40	40.0	MR	10–20	8.0
53.	D952CIMMYT	MS	20–30	24.0	S	40	40.0	MS	20–30	24.0
54.	SWW 1/904	MS	20–30	24.0	S	40	40.0	MS	20–30	24.0
55.	Euclide	MR	10–20	8.0	MS	20–30	24.0	MS	20–30	24.0
(St1) ^1^	Bogarnaya 56	S	60–80	80.0	-	-	-	-	-	-
(St2) ^2^	Morocco	-	-	-	S	40–80	80.0	-	-	-
(St3) ^3^	Bakytzhan	-	-	-	-	-	-	S	40–60	60.0

* IT—infection type (I—immune, R—resistant, MR—moderately resistant, MS—moderately susceptible, S—susceptible), ** DS—disease severity (%), *** CI—coefficient of infection, ^1, 2, 3^—Susceptible standards for leaf rust, yellow rust, and for stem rust, respectively.

**Table 2:** The results of the assessment of winter wheat cultivars to the yellow, leaf, stem rust races at the seedling stage and the identification of resistance genes.

Entry	Reaction Type to Rust Isolates at the Seedling Stage *															Identified Resistance Genes	Field Resistance **		
	Leaf Rust						Yellow Rust				Stem Rust						Leaf Rust	Yellow Rust	Stem Rust
	PtK1	PtK2	PtK3	PtK4	PtK5	PtK6	Pst_1	Pst_2	Pst_3	Pst_4	PgK1	PgK2	PgK3	PgK4	PgK5				
Adilet	1+	0; 1	3–4	3–4	3–4	3–4	2	3	3	3	2	2	3+	3	4	*Lr3* *Lr34* *Yr* *18 Sr57*	MR 20	MR 20	R 5–10
Almaly	4	3	3–4	3–4	3–4	3–4	4	4	3	3	3	3	4	3	3-	*Lr34* *Yr* *18 Sr57*	MR 10–20	MR 20	MR 10–20
Akhmat	1+	1	0–2	0–1	3–4	3–4	2	2	0–1;	2	3	2	4	4	3	*Lr1* 1Al.1RS	I-R 0–1	R 1–5	R 1–5
Amanat	3	2+	3–4	3–4	3–4	3–4	3	3	3	3	4	3	4	3	3	*-*	MS 20–30	S 40-60	MS 20–30
Bezostaya 100	0	0	0–1	0–1	0–1	0–1	0	0	3	3	2+	2+	2	1–2	1–2	*Lr26 Lr34 Yr9 Yr18 Sr31 Sr57*	I-R 0–1	R 1–10	I 0
D952CIMMYT	2	2	3–4	3–4	3–4	3–4	0	0	3	3	4	3+	4	4	4	*-*	MS 20–30	S 40	MS 20–30
Dulaty	3	3	3–4	3–4	3–4	3–4	3	3	3	2–3	3	4	3-	4	4	*Lr34 Yr* *18 Sr57*	MR 10–20	R-MR 5–10	MR 10–20
Egemen 20	2	3	3–4	3–4	3–4	3–4	2+	2	3	3	3	3	4+	3	4	*-*	MS-S 30–40	MS-S 30–40	MS 20–30
Euclide	2	1+	3–4	3–4	3–4	3–4	2	0	2–3	3	3	2	3-	3	3-	*Lr1*	MR 10–20	MS 20–30	MS 20–30
KIZ 90	0; 1	2	0–1	0;	0;	0;	2	0	3	3	0; 1	2-	2	1-2	2	*Lr1 Lr3 Lr26 Yr9 Sr31*	I 0	I-R 0–5	I 0
Steklovidnaya24	3	4	3–4	3–4	3–4	3–4	4	4	3	3	4	4	3	3	4	*-*	MS-S 30–40	MS-S 30–40	MS 20–30
SWW 1/904	0; 1	0	3–4	3–4	3–4	3–4	3	2	0–1;	3	3	2+	4	4	4	*-*	MS 20–30	S 40	MS 20–30
18410-1	0; 1	1+	0;	0–1;	0–1;	0–1;+	1	1	0;	0	0; 1	1	3	3-	3	*Lr34* *Yr* *18 Sr57*	R-MR 5–20	R-MR 5–20	MR 10–20
20197-17	2	2	3–4	3–4	3–4	3–4	1	2	2–3	3	3	3+	4	3	4	*Lr34* *Yr* *18 Sr57*	R-MR 5–20	R-MR 5–20	R-MR 5–20
20521-1	0	2	3–4	3–4	3–4	3–4	2	4	3	3	2	2	4+	3+	4	*-*	MS 30	S 40	S 40
21203-11-3	3	2	3–4	3–4	3–4	3–4	0	2	3	3	2	2	4	4	3	*Lr3*	MS 20–30	S 40	S 40
21730-1	1	0	0–1	0–1	3–4	3–4	0	3	3	3	0	0; 1	2	2+	1-2	*Lr3 Lr26 Yr9 Sr31*	R 5–10	I 0	I 0
22180-1	2	2	3–4	3–4	3–4	3–4	0	0	3	3	2+	3	3	4	4	*Lr34 Yr* *18 Sr57*	MR 10–20	MR 10–20	MR 10–20
22353K	2	1+	3–4	3–4	3–4	3–4	2	0	3	3	2	2	4	3+	4	*Lr1 Lr3 Lr34 Yr18 Sr57*	R-MR 1–20	MR 10–20	R 1–10
22372K	1+	1+, 2	0–1	0–1	3–4	3–4	0	0	3	3	1	2	2-	1–2	2-	*Lr3 Lr26 Yr9 Sr31*	R 1–5	I 0	I 0

* Reaction types for seedlings were 0, 0; 1, 2 for resistance and 3, 4 for susceptibility; «;» hypersensitive flecks; «+» more than average for the class. ** Rust severity in the field: infection type, IT (I—immune, R—resistant, MR—moderately resistant, MS—moderately susceptible, S—susceptible) and disease severity, DS (%).

**Table 3:** Precipitation and average air temperature from sowing to harvesting of winter wheat in the Almaty region in 2022–2024.

Year	Indicator	Months From Sowing to Harvesting of Winter Wheat									
		October	November	December	January	February	March	April	May	June	July
2021–2022	Precipitation, mm.	77.7	41.3	14.0	16.3	33.9	168.6	46.8	145.4	35.9	15.1
	Air temperature, °C	7.9	1.1	1.3	0.0	0.8	5.8	16.7	19.0	24.3	26.5
2022–2023	Precipitation, mm.	42.2	128.2	14.0	36.9	34.0	61.2	68.2	43.4	4.3	36.6
	Air temperature, °C	11.0	2.9	−4.6	−6.9	0.3	8.4	11.9	17.2	24.6	27.1
2023–2024	Precipitation, mm.	70.9	67.8	64.9	38.8	43.6	135.5	111.3	121.2	19.7	85.2
	Air temperature, °C	13.4	6.8	−1.0	−1.2	−4.0	5.4	12.8	17.6	24.5	25.0

**Table 4:** Virulence/avirulence profile of *P. triticina*, *P. striiformis*, and *P. graminis* isolates.

Isolate	Origination	Virulence to Genes	Avirulence to Genes
*Puccinia triticina*			
Pt_1_TGT	Kazakhstan, Kostanay, 2021	*Lr*: 1, 2a, 2b, 2c, 3a, 3bg, 3ka, 11, 14a, 16, 17, 20, 30	*Lr:* 9, 19, 24, 25, 26, 29
Pt_1_ KHT	Kazakhstan, Almaty, 2022	*Lr:* 2a, 2b, 2c, 3a, 3bg, 3ka, 11, 14a, 16, Lr17, 26, 30	*Lr:* 1, 9, 15, 19, 24, 25, 26, 29
PtK1	Russia, Chelyabinsk, 2022	*Lr:* 1, 2a, 2b, 2c, 3a, 3bg, 3ka, 9,10, 14a, 14b, 15, 17, 18, 20, 30	*Lr:* 19, 16, 24, 26, 28, 47, 51
PtK2	Russia, Saratov, 2021	*Lr:* 1, 2a, 2b, 2c, 3a, 3bg, 3ka, 10, 14a, 14b, 15, 16, 17, 18, 19, 20, 30	*Lr:* 9, 24, 26, 28, 29, 47, 51
PtK3	Russia, Novosibirsk, 2021	*Lr:* 1, 2a, 2b, 2c, 3a, 3bg, 3ka, 10, 14a, 14b, 15, 16, 17, 18, 20, 26, 30	*Lr:* 9, 19, 24, 28, 29, 47, 51
PtK4	Russia, Dagestan, 2023	*Lr:* 1, 2c, 3a, 3bg, 3ka, 10, 14a, 14b, 16, 17, 18, 20, 26, 30	*Lr:* 2a, 2b, 9, 15, 19, 24, 28, 29, 47, 51
*Puccinia striiformis* f. sp. *tritici*			
Pst_1	Kazakhstan, Taraz, 2022	*Yr:* 1, 6, 7, 8, 12, 18, 27	*Yr:* 5, 9, 10, SP, 26
Pst_2	Kazakhstan, Almaty, 2022	*Yr:* 1, 6, 8, 9, 18, 26	*Yr:* 5, 7, 10, 12, SP, 27
Pst_3	Russia, Novosibirsk, 2021	*Yr:* 1, 2, 3, 6, 8, 9, 27, SD	*Yr:* 4, 5, 7, 10, 15, 17, 24, SP, ND
Pst_4	Russia, St. Petersburg, 2022	*Yr:* 2, 3, 4, 6, 8, 9, 27	*Yr:* 1, 5, 7, 10, 15, 17, 24, SP, SD, ND
*Puccinia graminis* f. sp. *tritici*			
PgK1	Kazakhstan, Kostanay, 2021	*Sr:* 5, 6, 7b, 9a, 9d, 9g, 9e, 10, 17, 21, 36, 38, Tmp, McN	*Sr:* 8a, 9b, 11, 24, 30, 31
PgK2	Kazakhstan, Almaty, 2022	*Sr:* 5, 6, 9a, 9b, 9d, 9g, 10, 17, 21, 38, McN	*Sr:* 7b, 8a, 9e, 11, 24, 30, 31, 36, Tmp
PgK3	Russia, Saratov, 2022	*Sr:* 5, 21, 9e, 7b, 11, 6, 8a, 9g, 36, 9b, 30, 17, 9a, 9d, 10, Tmp, 38, McN	*Sr:* 24, 31
PgK4	Russia, Tatarstan, 2023	*Sr:* 5, 21, 9e, 7b, 11, 6, 8a, 9g, 36, 9b, 3, 17, 9a, 9d, 10, Tmp, 24, 38, McN	*Sr:* 31
PgK5	Russia, Saratov, 2022	*Sr:* 5, 21, 9e, 7b, 11, 6, 8a, 9g, 36, 9b, 30, 9a, 9d, 10, Tmp, 31, 38, McN	*Sr:* 17, 24, 31

**Table 5:** Molecular markers for the identification of the *Lr*, *Yr*, and *Sr* genes.

Gene	Marker	Allele Size, bp	References
*Lr1*	WR003 F/R	760	[65]
*Lr3a*	Xmwg798	365	[66]
*Lr9*	SCS5	550	[67]
*Lr10*	F1.2245/*Lr*10-6/r2	310	[68]
*Lr25*	*Lr*25F20/R19	1800	[69]
*Lr28*	SCS421	570	[70]
*Lr29*	*Lr*29F24	900	[69]
*Lr41 (39)*	GDM35	190	[71]
*Lr47*	PS10	282	[72]
*Lr51*	S30-13L/AGA7-759	783, 422	[73]
*Lr66 (Asp)*	S13-R16	695	[74]
*Lr19*, *Sr25*	SCS265	512	[75]
*Lr20*, *Sr15*	STS638	542	[76]
*Lr24*, *Sr24*	*Sr*24 ≠ 12, *Sr*24 ≠ 50	500, 200	[77]
*Lr26*, *Sr31*, *Yr9*	SCM9	207 (1BL.1RS), 228 (1AL.1RS)	[78]
*Lr34*, *Sr57*, *Yr18*	csLV34	150	[79]
*Lr37*, *Sr38*, *Yr17*	Ventriup/LN2	259	[80]
*Lr_Yr6Agi2*	MF2/MR1r2	347	[81]
*Sr2*	csSr2	172	[82]
*Sr28*	wPt-7004-PCR, Xwmc332	194, 214, 217, 220	[83]
*Sr26*	*Sr*26#43	207	[77]
*Sr36*	Xwmc477, Xstm773-2	190, 155	[84]

## Data Availability

The data presented in the study are available on request from the corresponding author.

## Supplementary Materials

The following supporting information can be downloaded at: https://www.mdpi.com/article/10.3390/plants14071146/s1, Table S1: Pedigrees and origin of cultivars and breeding lines of winter wheat (*Triticum aestivum* L.) taken for experiments. Table S2: Dynamics of leaf, yellow, and stem rust severity on winter wheat cultivars and breeding lines in 2024, Almaty region, Kazakhstan; Table S3: Meteorological data in the Almaty region of Kazakhstan for 2022, 2023, and 2024; Figure S1: Photos of the development of leaf, yellow, and stem rust on winter wheat cultivars and breeding lines in the Almaty region of Kazakhstan in 2024; Figure S2: Results of the molecular analysis of the study of *Lr*, *Yr*, and *Sr* resistance genes.
