# Supplementary material for: Characterizing the Genetic Basis of Winter Wheat Rust Resistance in Southern Kazakhstan
Source: Plants (Basel). 2025 Apr 7;14(7):1146. doi: 10.3390/plants14071146 (PMC11991061; doi:10.3390/plants14071146)
Supplement: Supplementary file 1 [file plants-14-01146-s001.zip › plants-3544023-supplementary.pdf]

Article

Characterizing the Genetic Basis of Winter Wheat Rust Resistance in Southern Kazakhstan

Table S1. Pedigrees and origin of cultivars and breeding lines of winter wheat (*Triticum aestivum* L.) taken for experiments.

No.	Entry	Pedigree	Origin
1.	Adilet	Bezostaya 1/*Ae.cylindrica*/T.kiharae/Zhadyra	Kazakhstan
2.	Almaly	K-50431, Bul./ Bezostaya 1	Kazakhstan
3.	Amanat	Zhetysu/Bassar	Kazakhstan
4.	Arap	Bezostaya 1/8735Durum// Bezostaya 1	Kazakhstan
5.	Bakytzhan	Steklovidnaya 24/Almaly	Kazakhstan
6.	Dimash	A.Atin.p.k./Dnepr.521	Kazakhstan
7.	Dulati	MK3677/Naz	Kazakhstan
8.	Egemen 20	Alm.6783-69/7 (K-50431/ Bezostaya 1)	Kazakhstan
9.	Farabi	Karlygash/Durum680	Kazakhstan
10.	Kazakhstansk.10	Priboy/Strela	Kazakhstan
11.	KIZ 90	Przhev./AD121-10//Almaly	Kazakhstan
12.	Mereke 70	Bogarnaya 56/Kavkaz// Bogarnaya 56/OPAKS1	Kazakhstan
13.	Momyshuly	Steklovidnaya 24/Almaly	Kazakhstan
14.	Nesipkhan	Zhalyn/Naz	Kazakhstan
15.	Pamyat 47	K298669/L10/130H2/320/B.1/47157	Kazakhstan
16.	Sapaly	Bogarnaya 56/Albidum114//Krupnokol.	Kazakhstan
17.	Steklovidnaya 24	Bogarnaya 56/Tepl.2//Rostovchanka	Kazakhstan
18.	Talimi 80	Taza/Mironovskaya ost.	Kazakhstan
19.	Vavilov	Mironovskaya 808/Obri	Kazakhstan
20.	Zhetysu	A.Atin.p.k./MVG-03	Kazakhstan
21.	18410-1	Zhetysu/Bogarnaya 56	Kazakhstan
22.	18411-1	Zhetysu/Albatross Odess.	Kazakhstan
23.	20197-17	Kin-4/Almaly	Kazakhstan
24.	20403-2	Rassad/Salamoom	Kazakhstan
25.	20521-1	19982/20064	Kazakhstan
26.	20933-1	Naz/MV Dalma	Kazakhstan
27.	20982-2	SWW1-97/Arap	Kazakhstan
28.	21144-4-1	Odess.120/20977	Kazakhstan
29.	21203-11-3	Almaly 1-15/18423-4	Kazakhstan
30.	21266-3	Zhetysu/SWW 2-126	Kazakhstan
31.	21730-1	(G-1782-125/Ubil.)/Mereke 75	Kazakhstan
32.	22180-1	Matai/Birusa	Kazakhstan
33.	22315-1	18421-4/3-203Sonmes	Kazakhstan
34.	22353K	P.47/D.24SA	Kazakhstan
35.	22372K	Steklovidnaya 24/D.42	Kazakhstan
36.	Alekseyich	Lut.2935к51/Fortuna	Russia
37.	Akhmat	L.252-91к11-1/Smuglianka	Russia
38.	Bezostaya 100	Lut.3415к8-6-4/Lut.198-93к80	Russia
39.	Grom	L.1171-95/L.2919к3	Russia
40.	Gurt	Tanya/Frontana	Russia
41.	Bardosh	SERI.1B//KAUZ/HEVO/3/AMAD	Uzbekistan
42.	Ezoz	38th ESWYT.64//PRLII/CM65531/3/...	Uzbekistan
43.	Ilgor	IBWSN.1291FPAU\SERI/1B\AMAD\...	Uzbekistan

44.	Kayraktosh	The ancient cultivar "Kairaki"	Uzbekistan
45.	Ok marvarid	Unumli bugdai/K5076 (SAFVIR)	Uzbekistan
46.	Pahlavon	4EF_Marzhon (Sanzar-85/K-47335)	Uzbekistan
47.	Tespishar	Bezostaya 1/ Grekum-646, Lines	Uzbekistan
48.	Ajara	Selection from the material of Kyrgyzstan	Kyrgyzstan
49.	Asyl	Don.bezostaya/Krasnovodopadskaya 210	Kyrgyzstan
50.	Intensivnaya	Bezostaya 1/ Kazakhstanskaya 126	Kyrgyzstan
51.	D68CIMMYT	OR943576/KS920705CBUNT-RN	CIMMYT
52.	D580CIMMYT	F5-425/Renan	CIMMYT
53.	D952CIMMYT	13IWWIT-IR(PEHLIVAN/Jagger)	CIMMYT
54.	SWW 1/904	CIMMYT, Super wheat	CIMMYT
55.	Euclide	France, Florimond Despres	France
		Susceptible standarts:	
(St)[1]	Bogarnaya 56	Jub.Oset/Oct.Trit.LV-1// Bezostaya 1	Kazakhstan
(St)[2]	Morocco	-	CIMMYT
(St)[3]	Bakytzhan	Steklovidnaya 24/Almaly	Kazakhstan

Table S2. Dynamics of leaf, yellow and stem rust severity on winter wheat cultivars and breeding lines in 2024, Almaty region, Kazakhstan.

No	Name	*P. triticina*				*P. striiformis*				*P. graminis*			
		IT*	DS**, 1 scoring	DS**, 2 scoring	DS**, 3 scoring	IT*	DS**, 1 scoring	DS**, 2 scoring	DS**, 3 scoring	IT*	DS**, 1 scoring	DS**, 2 scoring	DS**, 3 scoring
1.	Adilet	MR	10	20	20	MR	10	10	20	R	1	5	10
2.	Almaly	MR	10	10	20	MR	10	20	20	MR	10	10	20
3.	Amanat	MS	10	20	30	S	20	40	60	MS	10	20	30
4.	Arap	MS	10	10	30	MR-MS	10	20	30	S	20	30	40
5.	Bakytzhan	MS	10	20	30	MR-MS	10	20	30	S	20	40	60
6.	Dimash	MS	10	10	30	MR	10	10	20	MS	10	20	30
7.	Dulati	MR	10	10	20	R-MR	1	5	10	MR	10	10	20
8.	Egemen 20	MS-S	20	30	40	MS-S	20	20	40	MS	10	20	30
9.	Farabi	MS-S	20	30	40	MS	20	20	30	MS	10	20	30
10.	Kazakhstan 10	MS-S	10	20	40	MR-MS	10	20	30	MR-MS	10	20	30
11.	KIZ90	I	0	0	0	I-R	0	0	5	I	0	0	0
12.	Mereke 70	R-MR	5	10	20	R	1	5	10	MR-MS	10	20	30
13.	Momyshuly	MS	10	20	30	MR-MS	10	20	30	MR-MS	10	20	30
14.	Nesipkhan	MS	10	10	30	MR	10	10	20	MR-MS	10	20	30
15.	Pamyat 47	MS	10	20	30	S	40	60	80	MR-MS	10	20	30
16.	Sapaly	MR-MS	10	20	30	MR-MS	10	20	30	R-MR	1	5	10
17.	Steklovidnaya 24	MS-S	20	30	40	MS-S	20	30	40	MS	20	30	30
18.	Talimi 80	MR-MS	10	20	30	MR	10	10	20	MR	10	10	20
19.	Vavilov	MS-S	20	30	40	MR-MS	10	20	30	MR-MS	10	20	30
20.	Zhetysu	MS-S	20	30	40	MS-S	20	30	40	MS	20	20	30
21.	18410-1	R-MR	5	10	20	R-MR	5	10	20	MR	10	10	20
22.	18411-1	MS	10	20	30	MS-S	20	30	40	R	1	5	10
23.	20197-17	R-MR	5	10	20	R-MR	5	10	20	R-MR	5	10	20
24.	20403-2	I	0	0	0	MR-MS	10	20	30	R	1	1	5
25.	20521-1	MS	10	20	30	S	20	30	40	S	20	30	40
26.	20933-1	MS	10	20	30	MR-MS	10	20	30	R-MR	5	10	20
27.	20982-2	MS	10	20	30	R-MR	1	5	10	MR	10	10	20
28.	21144-4-1	R	1	5	10	MR-MS	10	20	30	R-MR	1	5	10
29.	21203-11-3	MS	10	20	30	S	20	30	40	S	20	30	40
30.	21266-3	MR	10	10	20	MR	10	10	20	R-MR	1	5	10

No	Name	*P. triticina*				*P. striiformis*				*P. graminis*			
		IT*	DS**, 1 scoring	DS**, 2 scoring	DS**, 3 scoring	IT*	DS**, 1 scoring	DS**, 2 scoring	DS**, 3 scoring	IT*	DS**, 1 scoring	DS**, 2 scoring	DS**, 3 scoring
31.	21730-1	R	1	5	10	I	0	1	0	I	0	0	0
32.	22180-1	MR	10	10	20	MR	10	10	20	MR	10	10	20
33.	22315-1	R	1	5	10	MR-MS	20	20	30	I-R	0	0	5
34.	22353K	R-MR	5	10	20	MR	20	20	30	R	1	1	10
35.	22372K	R	1	1	5	I	0	0	0	I	0	0	0
36.	Alekseyich	R	1	5	10	I-R	0	0	5	R	1	1	5
37.	Akhmat	I-R	0	0	1	R	1	1	5	R	1	1	5
38.	Bezostaya 100	I-R	0	0	1	R	1	1	10	I	0	0	0
39.	Grom	R	1	5	10	MR	10	10	20	MS	20	20	30
40.	Gurt	MS	10	20	30	S	40	60	80	MR	10	10	20
41.	Bardosh	MS	10	20	30	MR	5	10	20	MR-MS	10	20	30
42.	Ezoz	R	1	5	10	S	40	60	80	MR-MS	10	20	30
43.	Ilgor	R	1	5	10	R	1	5	10	MR-MS	10	20	30
44.	Kayraktosh	R	1	5	10	MS-S	20	30	60	MR-MS	10	20	30
45.	Ok marvarid	MS-S	20	30	40	MS-S	20	30	40	MR-MS	10	20	30
46.	Pahlavon	R-MR	5	10	20	MS-S	20	30	40	MR-MS	10	20	30
47.	Tespishar	MS	10	20	30	S	40	60	80	MR	10	10	20
48.	Ajara	MR	5	10	20	S	40	60	80	MR-MS	10	20	30
49.	Asyl	MR	5	10	20	MR-MS	10	20	30	MR-MS	10	20	30
50.	Intensivnaya	MS-S	30	40	60	MS-S	20	30	40	MR-MS	10	20	30
51.	D68CIMMYT	R	1	5	10	S	20	30	40	R-MR	1	5	10
52.	D580CIMMYT	MS-S	20	40	60	S	20	30	40	MR	10	10	20
53.	D952CIMMYT	MS	10	20	30	S	20	30	40	MS	20	20	30
54.	SWW 1/904	MS	10	20	30	S	20	30	40	MS	10	20	30
55.	Euclide	MR	5	10	20	MS	10	20	30	MS	20	20	30
(St)[1]	Bogarnaya 56	S	20	60	80	-	-	-	-	-	-	-	-
(St)[2]	Morocco	-	-	-	-	S	40	60	80	-	-	-	-
(St)[3]	Bakytzhan	-	-	-	-	-	-	-	-	S	20	40	60

*IT – Infection Type (I – Immune, R – Resistant, MR – Moderately Resistant, MS – Moderately Susceptible, S – Susceptible);
**DS – Disease Severity (%), For *Pt* – 1 scoring – 1/06/2024, 2 scoring – 11/06/2024, 3 scoring – 22/06/2024; For *Pst* – 1 scoring – 24/05/2024, 2 scoring – 5/06/2024, 3 scoring – 16/06/2024; For *Pgt* – 1 scoring – 18/06/2024, 2 scoring – 1/07/2024, 3 scoring – 12/07/2024.

Table S3. Meteorological data in the Almaty region of Kazakhstan for 2022, 2023 and 2024.

Month	10 days	Air temperature, °C					Precipitation, mm		
		Daily Average	Max.	Min.	Annual average	Temperature difference	Actual	Annual average	Precipitation difference
**Weather data for 2022**									
January, 2022	I	1.2	3.5	-1.0	-11.3	12.5	1.1	5.2	-4.1
	II	1.5	5.3	-1.1	-11.6	13.1	10.7	5.8	4.9
	III	-2.6	1.0	-4.8	-9.5	6.9	4.5	8.8	-4.3
	Per month	0.0	3.3	-2.3	-10.8	10.8	16.3	19.8	-3.5
February, 2022	I	-2.2	1.6	-4.6	-9.3	7.1	17.1	8.4	8.7
	II	-3.5	1.7	-6.8	-9.2	5.7	16.8	7.8	9.0
	III	3.3	9.6	-1.1	-7.0	10.3	0.0	5.7	-5.7
	Per month	0.8	4.3	-4.2	-8.5	9.3	33.9	21.9	12.0
March, 2022	I	6.9	10.4	3.4	-3.1	10.0	31.1	15.8	15.3
	II	5.1	8.3	3.1	0.8	4.3	66.6	13.0	53.6
	III	5.4	10.2	1.3	4.2	1.2	70.9	20.0	50.9
	Per month	5.8	9.6	2.6	0.7	5.1	168.6	48.8	119.8
April, 2022	I	17.2	23.5	11.3	7.9	9.3	6.5	16.4	-9.9
	II	17.5	23.8	10.5	10.9	6.6	5.3	21.6	-16.3
	III	15.4	21.3	10.2	12.2	3.2	35.0	18.4	16.6
	Per month	16.7	22.8	10.6	10.4	6.3	46.8	56.5	-9.7
May, 2022	I	20.5	26.6	14.9	15.8	4.7	13.7	18.7	-5.0
	II	18.5	24.3	12.9	16.0	2.5	61.3	22.7	38.6
	III	17.9	23.0	13.4	17.4	0.5	70.4	20.2	50.2
	Per month	19.0	24.6	13.7	16.4	2.6	145.4	61.6	83.8
June, 2022	I	22.4	28.3	16.8	20.3	2.1	4.8	24.4	-19.6
	II	23.9	30.3	17.7	21.2	2.7	6.2	16.1	-9.9
	III	26.7	32.3	20.1	22.1	4.6	24.9	13.4	11.5
	Per month	24.3	30.3	18.2	21.2	3.1	35.9	53.9	-18.0
July, 2022	I	24.9	30.1	19.1	23.5	1.4	6.2	10.8	-4.6
	II	25.7	32.4	19.1	23.7	2.0	0.6	8.8	-8.2
	III	28.9	35.4	21.9	25.0	3.9	8.3	7.0	1.3
	Per month	26.5	32.6	20.0	24.1	2.4	15.1	26.6	-11.5
August, 2022	I	24.6	31.7	19.1	24.0	0.6	7.3	8.7	-1.4
	II	23.1	30.5	17.2	22.6	0.5	0.9	13.8	-12.9
	III	20.3	27.6	13.7	22.1	-1.8	0.0	12.5	-12.5
	Per month	22.6	30.0	16.7	22.8	-0.2	8.2	35.4	-27.2
September, 2022	I	25.8	33.5	18.7	20.3	5.5	0.0	6.6	-6.6
	II	17.6	25.4	10.5	17.4	0.2	2.1	9.0	-6.9
	III	19.8	26.1	14.5	12.6	7.2	0.0	10.1	-10.1
	Per month	21.1	28.3	14.6	16.7	4.4	2.1	25.9	-23.8
October, 2022	I	12.4	19.6	5.8	13.2	-0.8	2.1	14.5	-12.4
	II	12.7	20.1	6.9	10.2	2.5	17.6	19.6	-2.0
	III	8.0	14.1	3.7	8.1	-0.1	22.5	10.2	12.3
	Per month	11.0	17.9	5.5	10.5	0.5	42.2	43.6	-1.4
November, 2022	I	6.5	11.3	3.0	5.1	1.4	37.4	12.3	25.1
	II	2.6	7.9	-1.0	1.9	0.7	16.7	18.9	-2.2

Month	10 days	Air temperature, °C					Precipitation, mm		
		Daily Average	Max.	Min.	Annual average	Temperature difference	Actual	Annual average	Precipitation difference
	III	-0.2	3.7	-3.3	0.3	-0.5	74.1	17.7	56.4
	Per month	2.9	7.6	-0.4	2.4	0.5	128.2	49.0	79.2
December, 2022	I	-5.4	-2.8	-7.2	-3.8	-1.6	7.4	16.7	-9.3
	II	-5.2	-1.0	-8.8	-7.6	2.4	0.0	11.1	-11.1
	III	-3.2	1.7	-6.0	-3.6	0.4	6.6	17.6	-11.0
	Per month	-4.6	0.7	-7.3	-4.9	0.3	14.0	45.3	-31.3
**Average / Amount for 2022**	**Per year**	**12.1**	**17.6**	**7.3**	**8.4**	**3.7**	**656.7**	**488.3**	**168.4**
**Weather data for 2023**									
January, 2023	I	-0.4	4.9	-2.8	-6.1	5.7	29.5	16.4	13.1
	II	-13.4	-7.7	-16.3	-7.4	-6.0	7.4	11.9	-4.5
	III	-6.8	-0.8	-10.7	-7.9	1.1	0.0	13.1	-13.1
	Per month	-6.9	-1.2	-9.9	-7.2	0.3	36.9	41.4	-4.5
February, 2023	I	0.0	4.8	-3.4	-6.4	6.4	13.5	14.5	-1.0
	II	-1.8	3.3	-4.6	-6.4	4.6	8.6	16.3	-7.7
	III	2.8	8.5	-1.5	-3.9	6.7	11.9	11.1	0.8
	Per month	0.3	5.5	-3.2	-5.1	5.4	34.0	41.9	-7.9
March, 2023	I	8.9	15.3	3.7	3.7	5.2	20.7	13.7	7.0
	II	6.1	12.3	2.1	2.3	3.8	38.8	26.1	12.7
	III	10.3	16.5	4.5	7.2	3.1	1.7	25.8	-24.1
	Per month	8.4	14.7	3.4	4.4	4.0	61.2	65.6	-4.4
April, 2023	I	9.9	16.2	3.9	8.4	1.5	14.0	30.7	-16.7
	II	10.9	16.4	5.8	11.9	-1.0	50.3	35.7	14.6
	III	14.9	21.9	8.6	14.1	0.8	3.9	42.1	-38.2
	Per month	11.9	18.2	6.1	11.5	0.4	68.2	108.5	-40.3
May, 2023	I	13.0	19.0	6.9	15.0	-2.0	4.2	29.6	-25.4
	II	20.4	26.6	15.1	16.9	3.5	10.8	24.1	-13.3
	III	18.2	26.6	15.1	18.6	-0.4	28.4	25.4	3.0
	Per month	17.2	23.0	11.5	16.8	0.4	43.4	79.1	-35.7
June, 2023	I	25.5	31.5	19.1	20.4	5.1	0.0	25.7	-25.7
	II	24.9	31.3	19.1	21.5	3.4	1.1	18.7	-17.6
	III	23.5	29.4	17.8	22.3	1.2	3.2	14.0	-10.8
	Per month	24.6	30.7	18.7	21.4	3.2	4.3	58.4	-54.1
July, 2023	I	23.7	30.3	17.8	23.7	0.0	14.9	18.4	-3.5
	II	29.4	35.9	23.1	24.0	5.4	0.0	72.2	-72.2
	III	28.2	35.1	21.7	23.9	4.3	18.7	14.1	4.6
	Per month	27.1	33.8	20.9	23.9	3.2	33.6	104.7	-71.1
August, 2023	I	28.1	34.6	22.4	24.0	4.1	12.8	8.7	4.1
	II	22.9	28.3	16.2	22.6	0.3	31.0	13.8	17.2
	III	23.7	29.4	17.5	22.1	1.6	29.1	12.5	16.6
	Per month	24.9	30.8	18.7	22.8	2.1	72.9	35.0	37.9
September, 2023	I	20.2	26.0	14.3	20.3	-0.1	4.6	6.6	-2.0
	II	17.3	22.5	13.4	17.4	-0.1	32.1	9.0	23.1
	III	15.7	20.4	9.9	12.6	3.1	23.1	10.1	13.0

Month	10 days	Air temperature, °C					Precipitation, mm		
		Daily Average	Max.	Min.	Annual average	Temperature difference	Actual	Annual average	Precipitation difference
	Per month	17.7	23.0	12.5	16.7	1.0	59.8	25.7	34.1
October, 2023	I	15.4	21.8	9.2	13.2	2.2	6.2	14.5	-8.3
	II	12.0	19.0	6.1	10.2	1.8	38.6	19.6	19.0
	III	12.8	18.8	7.4	8.1	4.7	26.1	10.2	15.9
	Per month	13.4	19.9	7.6	10.5	2.9	70.9	44.3	26.6
November, 2023	I	7.2	13.3	3.2	5.1	2.1	22.9	12.3	10.6
	II	6.9	13.1	2.8	1.9	5.0	17.9	18.9	-1.0
	III	6.3	12.7	2.6	0.3	6.0	27.0	17.7	9.3
	Per month	6.8	13.0	2.9	2.4	4.4	67.8	48.9	18.9
December, 2023	I	3.0	7.6	-0.1	-3.8	6.8	8.3	16.7	-8.4
	II	-7.8	-3.4	-11.0	-7.6	-0.2	20.1	11.1	9.0
	III	1.9	7.5	-1.4	-3.6	5.5	36.5	17.6	18.9
	Per month	-1.0	3.9	-4.2	-4.9	3.9	64.9	45.4	19.5
**Average / Amount for 2023**	**Per year**	**12.0**	**18.0**	**7.1**	**9.4**	**2.6**	**617.9**	**698.9**	**-81.0**
**Weather data for 2024**									
January, 2024	I	2.8	8.2	-0.5	-5.9	8.7	0.3	16.4	-16.1
	II	-2.2	2.7	-5.6	-7.7	5.5	31.3	11.9	19.4
	III	-4.2	-0.3	-6.9	-8.0	3.8	7.2	13.0	-5.8
	Per month	-1.2	3.5	-4.3	-7.4	6.2	38.8	41.3	-2.5
February, 2024	I	-0.7	5.2	-4.5	-6.3	5.6	6.1	16.1	-10.0
	II	-5.0	2.2	-10.0	-6.4	1.4	32.2	17.4	14.8
	III	-6.3	-0.5	-9.7	3.4	-9.7	5.3	11.1	-5.8
	Per month	-4.0	-2.3	-8.1	-5.3	1.3	43.6	44.6	-1.0
March, 2024	I	1.1	6.3	-2.9	3.7	-2.6	2.7	13.7	-11.0
	II	4.5	11.2	-1.5	2.3	2.2	46.6	26.1	20.5
	III	10.7	15.7	6.6	7.2	3.5	86.2	25.8	60.4
	Per month	5.4	11.1	0.7	4.4	1.0	135.5	65.6	69.9
April, 2024	I	11.2	16.7	5.9	8.4	2.8	37.4	31.6	5.8
	II	13.7	18.6	8.9	11.9	1.8	38.8	36.6	2.2
	III	13.5	18.1	9.0	14.1	-0.6	35.1	42.4	-7.3
	Per month	12.8	17.8	7.9	10.9	1.9	111.3	110.6	0.7
May, 2024	I	18.0	24.0	12.4	14.5	3.5	31.6	41.5	-9.9
	II	18.4	24.1	13.6	16.9	1.5	64.0	24.1	39.9
	III	16.3	22.6	11.1	18.6	-2.3	25.6	32.8	-7.2
	Per month	17.6	23.6	12.4	16.3	1.3	121.2	98.4	22.8
June, 2024	I	22.9	28.8	16.7	20.4	2.5	3.0	19.9	-16.9
	II	23.8	29.9	17.9	21.5	2.3	4.0	19.8	-15.8
	III	26.9	32.5	21.8	22.3	4.6	11.0	20.0	-9.0
	Per month	24.5	30.4	18.8	21.2	3.3	19.7	59.9	-40.2
July, 2024	I	22.6	28.8	16.8	23.7	-1.1	57.0	18.4	38.6
	II	24.0	30.6	18.3	24.0	0.0	27.4	72.2	-44.8
	III	28.3	34.4	22.2	23.9	4.4	0.8	14.1	-13.3
	Per month	25.0	31.3	19.1	23.7	1.3	85.2	56.9	28.3

Month	10 days	Air temperature, °C					Precipitation, mm		
		Daily Average	Max.	Min.	Annual average	Temperature difference	Actual	Annual average	Precipitation difference
August, 2024	I	28.9	34.8	22.9	24.0	4.9	0.0	8.7	-8.7
	II	25.8	31.5	20.2	22.7	3.1	17.0	13.8	3.2
	III	22.9	29.1	16.5	22.1	0.8	8.1	12.5	-4.4
	Per month	25.9	31.8	19.9	22.9	3.0	25.1	34.8	-9.7
September, 2024	I	16.7	23.7	11.9	19.5	-2.8	21.9	6.8	15.1
	II	13.0	21.9	8.8	17.5	-4.5	7.4	9.0	-1.6
	III	15.6	21.4	10.0	15.6	0.0	13.5	10.1	3.4
	Per month	15.1	22.3	10.2	20.9	-5.8	14.3	25.9	-11.6
October, 2024	I	16.7	23.7	10.7	12.8	3.9	0.6	14.5	-13.9
	II	9.4	14.2	5.2	10.0	-0.6	46.2	19.6	26.6
	III	9.8	15.0	5.3	8.1	1.7	25.0	10.2	14.8
	Per month	12.0	17.6	7.1	10.3	1.7	71.8	43.6	28.2
November, 2024	I	8.4	14.0	4.6	5.1	3.3	32.5	12.3	20.2
	II	3.5	9.4	-0.2	2.0	1.5	16.8	18.9	-2.1
	III	3.3	9.2	-0.8	-0.8	4.1	8.8	16.7	-7.9
	Per month	1.5	10.9	1.2	2.0	-0.5	58.1	47.9	10.2
December, 2024	I	-2.1	0.6	-4.3	-2.7	0.6	17.2	16.7	0.5
	II	-4.8	0.9	-8.3	-4.6	-0.2	27.2	11.5	15.7
	III	-3.2	2.5	-7.1	-5.8	2.6	1.1	17.8	-16.7
	Per month	-3.3	1.3	-6.6	-4.4	1.1	45.5	46.1	-0.6
**Average / Amount for 2024**	**Per year**	**11.2**	**16.9**	**6.5**	**9.7**	**1.5**	**770.1**	**675.6**	**94.5**

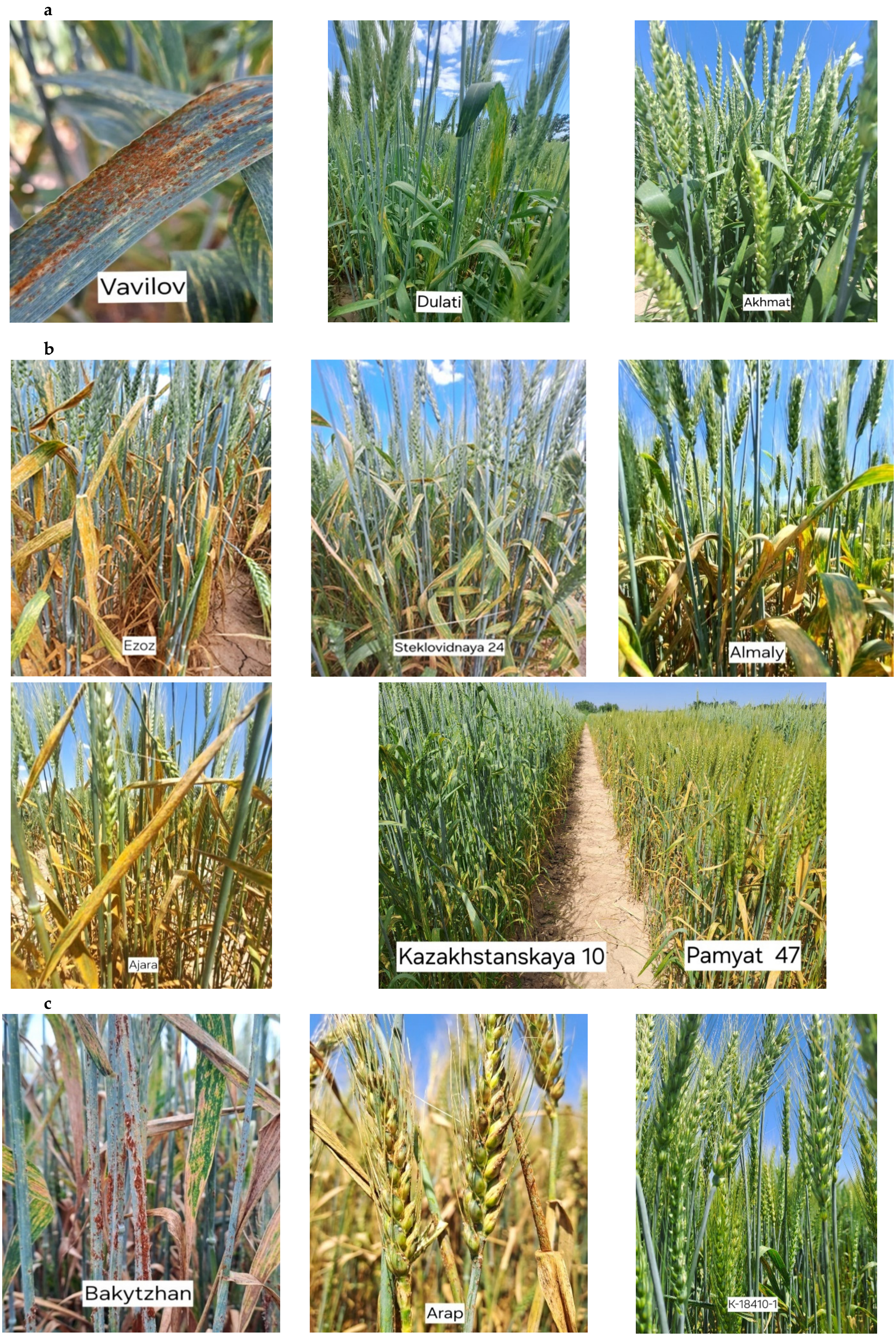

Figure S1. Photos of the development of leaf, yellow and stem rust on winter wheat cultivars and breeding lines in the Almaty region of Kazakhstan in 2024. **a.** Development of leaf rust on winter wheat cultivars; **b.** Development of yellow rust on winter wheat cultivars; **c.** Development of stem rust on winter wheat cultivars.

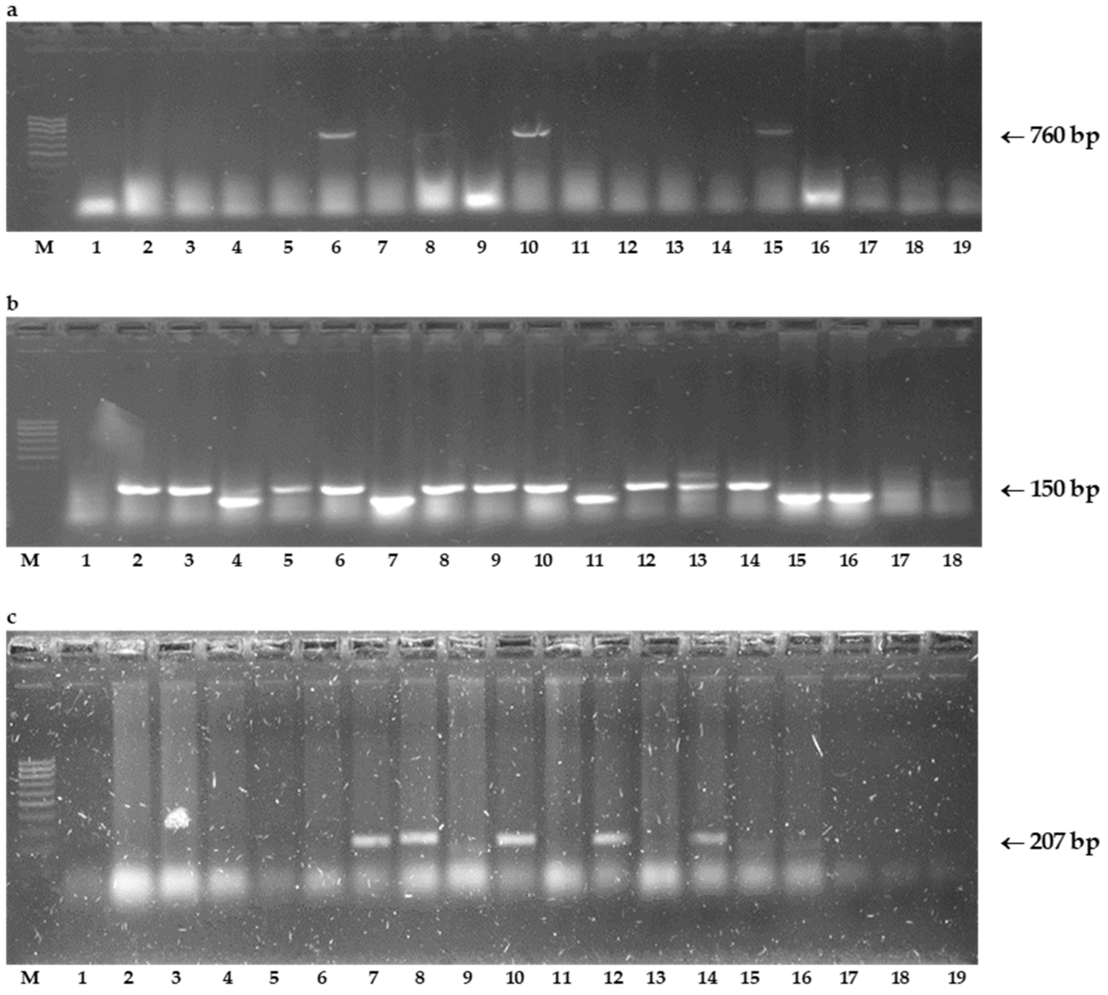

Figure S2. Results of the molecular analysis of the study of *Lr*, *Yr* and *Sr* resistance genes. Electrophoretogram for markers: **a**) WR003 (*Lr1*), **b**) csLV34 (*Lr34*/*Yr18*/*Sr57*/*Pm38*), **c**) SCM9 (*Lr26*/*Yr9*/*Sr31*/*Pm8*, 1AL.1RS). M, - DNA Ladder 100 bp (Dialat), 1, Almaly; 2, Steklovidnaya 24; 3, Egemen 20; 4, Dulati; 5., Amanat; 6, Euclide; 7, Bezostaya 100; 8, Akhmat; 9, SWW 1/904; 10, KIZ 90; 11, Adilet; 12, 21730-1; 13, 20521-1; 14, 22372K; 15, 22353K; 16, 18410-1; 17, 20197-17; 18, 21203-11-3; 19, D952CIMMYT.
